# Assessment of the color of orange juice in the context of dietitians’ food preferences

**DOI:** 10.3389/fnut.2023.1328795

**Published:** 2024-01-11

**Authors:** Marek Kardas, Agata Kiciak, Kamila Szynal, Barbara Sitkiewicz, Wiktoria Staśkiewicz-Bartecka, Agnieszka Bielaszka

**Affiliations:** ^1^Department of Food Technology and Quality Evaluation, Department of Dietetics, Faculty of Public Health in Bytom, Medical University of Silesia in Katowice, Zabrze, Poland; ^2^Doctoral School of the Medical University of Silesia in Katowice, Department of Human Nutrition, Faculty of Public Health in Bytom, Medical University of Silesia in Katowice, Zabrze, Poland

**Keywords:** color, dietitian, food preference, dietary choice, spectrophotometer, juice, consumer preference

## Abstract

**Introduction:**

Color is an integral part of product selection and is used to assess its attractiveness and quality. Dietitians are a group that influences the dietary choices of the population through education and promotion of rational eating behavior. The purpose of this study was to evaluate the color of selected juices in the context of dietitians’ food preferences.

**Methods:**

In the first stage of the research, the color of orange juices was measured using a spectrophotometer. In the second stage, sensory analysis was carried out using the ranking method. Participants were asked to assess the attractiveness of the color of juices through glasses and bottles without the original label and with the label. The juice with the best color turned out to be the juice which, according to the L * a * b * parameters, was relatively dark and had an intense orange tint.

**Results:**

As the juice with the worst color, they chose the juice that was colored green and blue. When assessing the color without and with the original label, the respondents indicated which one was significantly brighter and more yellow compared to the others. Dietitians prefer bright juices with a vibrant orange hue. Product packaging influences dieticians’ choices regardless of the content.

**Discussion:**

Instrumental control of color during product production and selection of packaging elements for attractive synergy are determinants of the perceived attractiveness of juices in the study group.

## 1 Introduction

Orange juices are popular products on the food market in Poland and Europe, not only because of their taste but also because of the health benefits resulting from the content of vitamins, minerals, and antioxidants. According to World Health Organization (WHO) recommendations, a glass of such juice can replace one portion of vegetables and/or fruit on a given day. Nutritionists who promote healthy foods may lean toward juices with colors associated with the maturity and quality of raw materials. Dietitians’ preferences for juice color stem from their understanding that color is related to nutrient content and influences sensory perception, which can affect fluid intake and the overall nutritional value of the product. Dietitians are the people who influence and modify their patients’ food choices, so the aspect of evaluating their consumer preferences is important (1–3). So, what makes a dietitian prefer a particular brand of juice?

Sensory senses such as taste, smell, texture, and color play an important role in the choice of a specific product. In addition to the brand, price, and type of packaging, the most important sense determining its purchase is the sense of sight. It provides information about the size, shape, and color of the product, which proves its freshness and attractiveness (1, 2). The visual appearance of food in terms of color has a great influence on the perception of food quality. Color may be correlated with other quality features, such as sensory, nutritional, and visual or non-visual defects, and its changes enable them to be controlled immediately (3, 4).

Integrating dieters’ preferences with CIELAB’s color assessment allows for a holistic analysis, combining health aspects with precise and objective color measurements, which can be important both for effective nutrition communication and for maintaining the high sensory quality of food products (5).

CIELAB is one of the most common color spaces for measuring the color of objects and is widely used in the color control and management industry (6). The CIELAB method, an international standard in color analysis, enjoys widespread use in scientific research on fruit juice color because of its standardization and accurate representation of human color perception. The CIELAB color space is based on a three-coordinate system: L * (brightness), a * (green-red tones), and b * (blue-yellow tones), enabling precise quantification of color intensity, brightness, and hue. Its international nature facilitates the comparability of measurement results between different laboratories on a global scale. The adoption of this method also stems from its consideration of human color perception, making it particularly useful in the context of sensory analysis of food products such as fruit juices. The choice of CIELAB stems from its ability to directly address fundamental aspects of color perception, such as brightness, saturation, and hue. In addition, the CIELAB space allows monitoring of color changes over time, which is crucial for food products subject to natural oxidation or enzymatic browning processes. Thanks to the intuitive nature of CIELAB coordinates, measurement results are easy to understand and interpret, facilitating analysis and data presentation in the context of scientific research on fruit juice colors (7, 8).

Sensory analysis techniques are also used to optimize sales, which show the preferences of potential consumers about the examined factor (1, 9). One of these methods is the scheduling method. It is an intermediate between the differential methods and the scaling methods. It consists of arranging several samples, given in random order, in terms of the selected quality feature (e.g., from the worst to the best color). The scale of the difficulty of the task depends on the size of the differences between the samples. Its advantages are simple tasks and speed of the assessment (10).

Packaging at the point of sale is the manufacturer’s last chance to convince the customer of their product. Research suggests that the inclusion of shape and color elements in the packaging design may affect the perception of quality by consumers, their emotional reactions related to the brand/product, and, consequently, general preferences (11–13). Moreover, the sensory stimuli contained in the packaging help to distract the customer from routine purchasing activities, attracting their attention through novelty and mediating product categorization (14).

The main objective of the study was to evaluate the color of selected food products in the context of dieters’ food preferences. The specific objectives were the measurement of the color of selected orange juices using the spectrophotometric method in the CIELAB color system, the consumer assessment of the attractiveness of selected orange juices using the scheduling method, and the assessment of the relationship between the appearance of the packaging and the consumer assessment of the color of selected orange juices.

## 2 Materials and methods

### 2.1 Study design

The research covered six pasteurized orange juices (100% juice) from various producers (sample designations: RJ, VF, LI, SN, SO, and ON), available on the Polish market. The study was carried out in a sensory analysis laboratory designed according to the PN-EN ISO 8589: 2017 standard. The Declaration of Helsinki of the World Medical Association guided the conduct of this study. The study protocol (KNW-0022/KB1/73/I/16) was reviewed by the Bioethics Committee of the Silesian Medical University in Katowice and was approved. Each person participating in the study gave informed consent to participate in the study and was informed about the anonymity of the results.

### 2.2 Color measurements

Color measurements were made with a Tri-Color SF80 spectrophotometer calibrated according to the L * 90.08, a * −0.74, and b * 0.70 standards, in the SCI3 measurement geometry, with a D654 light source and an observation angle of 10°. Further, 30 ml of a given orange juice was poured into a cuvette to measure the liquid and 15 measurements were made, from which the arithmetic mean was then calculated. Before measuring the color of a juice from another manufacturer, the cuvette was washed under running water and wiped dry.

### 2.3 Sensory analysis

Sensory analysis was conducted among 71 female dietetics students at the Medical University of Silesia in Katowice in the laboratory of sensory analysis. All the students were active in the dietetics profession. The analysis was carried out in three stages. In the first station, the color was assessed through 50 ml plastic glasses with 30 ml of juice. On the second stand, the juice was assessed through the original bottle without the manufacturer’s label. At the last stand, the color of the juice was assessed through the original bottle with the manufacturer’s label. The samples were numbered with three-digit random numbers. The evaluation was performed using the scheduling method prepared by the PN-ISO 5497: 1998 standard.

### 2.4 Statistical analysis

The results of color measurements were compiled using Color QC and Microsoft Office Excel 2019.

Statistical calculations were made in Statistica 13.3 (StatSoft 2017). To statistically analyze the results of the conducted sensory analysis using the scheduling method, the Friedman test was used and the Kendall coefficient of concordance was used. To calculate the correlation between the studied discriminants and the significance of differences between the mean values, the one-way ANOVA and a *post-hoc* test, the method of least significant differences (NIR), were used.

A correlation analysis was conducted between rank scores and color measurements in CIELAB space. Pearson’s r correlation coefficients were used to determine whether there is a relationship between the two variables.

The criterion for statistical significance was *p* < 0.05.

## 3 Results

### 3.1 Instrumental analysis of orange juice color using the spectrophotometric method in the CIELAB color system

The RJ orange juice color measurements in terms of the L * parameter ranged from 39.77 to 39.93. In terms of the a * parameter, the values were from −0.39 to −0.42, and in the case of the b * parameter, the values were from 15.75 to 15.90.

The L * parameter measurements for VF orange juice ranged from 39.32 to 39.35. For the a * parameter, the values were from −1.40 to −1.44, and for the b * parameter, the values were from 12.95 to 13.00.

For LI orange juice, the color measurements in terms of the L * parameter ranged from 40.77 to 40.98. In terms of the a * parameter, the values were from −1.37 to −1.52, and in the case of the b * parameter, the values were from 12.70 to 13.03.

SN orange juice obtained the following measurement results: parameter L * values between 43.14 and 43.37; parameter a * values from −0.46 to −0.49; parameter b * values from 15.81 to 16.05.

The SO orange juice color measurements in terms of the L * parameter ranged from 43.89 to 44.09. In terms of the a * parameter, the values were from −0.88 to −0.94, and for the b * parameter, the values were from 17.68 to 17.86.

For ON orange juice, the color measurements in terms of the L * parameter ranged from 41.13 to 41.33. In terms of the a * parameter, the values were from −0.58 to −0.62, and for the b * parameter, the values were from 14.99 to 15.05.

Comparing the arithmetic means of the measurements, the orange juice SO had the highest L * value and VF juice the lowest. In turn, the highest value of a * was for SN juice and the lowest LI. SO juice showed the highest value of the b * parameter and LI juice had the lowest (Table 1).

**TABLE 1:** Comparison of the mean values of the measurements.

Juice name	L * (*$\bar{x}$* ± SD)	a * (*$\bar{x}$* ± SD)	b * (*$\bar{x}$* ± SD)	*p*
RJ	39.87 ± 0.04	−0.41 ± 0.01	15.83 ± 0.04	0.13
VF	39.34 ± 0.01	−1.41 ± 0.01	12.97 ± 0.02	0.02*
LI	40.92 ± 0.07	−1.47 ± 0.04	12.79 ± 0.1	0.33
SN	43.32 ± 0.06	0.47 ± 0.01	15.91 ± 0.07	0.12
SO	43.98 ± 0.05	−0.92 ± 0.02	17.77 ± 0.07	0.12
ON	41.23 ± 0.04	−0.59 ± 0.01	15.03 ± 0.02	0.04*

L *, brightness; a *, green-red tones; b *, blue-yellow tones; $\bar{x}$, average; SD, standard deviation.

*p < 0.05.

**FIGURE 1:**
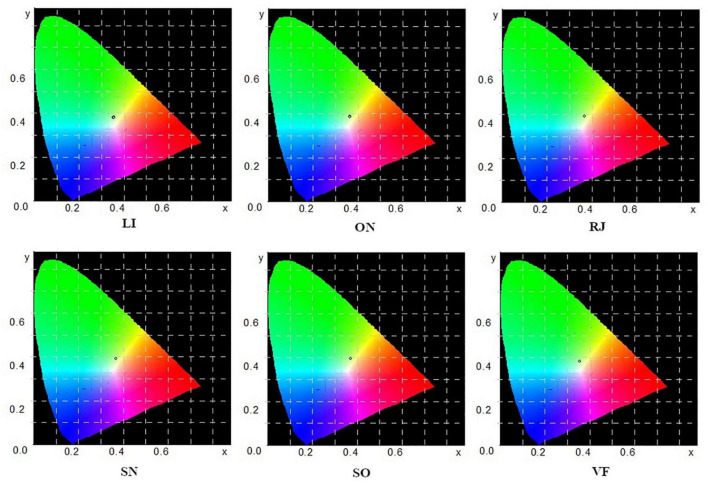
Juice color measurement values are located on chromaticity charts.

The average values of juice color measurement are also presented in the chromaticity graphs (Figure 1).

**FIGURE 2:**
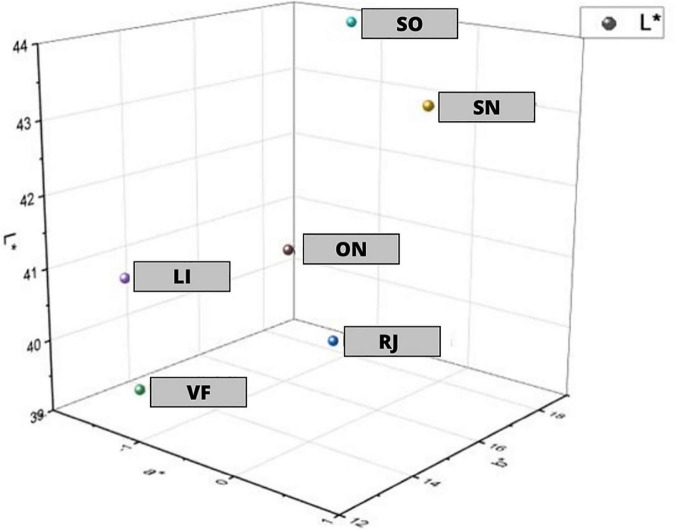
Juice color in the color space in the CIE L*a*b* system.

Based on these results, it can be concluded that SO juice was the brightest and that its color deviated the most toward yellow. On the other hand, the juice values differ the most between green and blue in comparison to other juices. VF juice was the darkest among the subjects and SN juice was the closest to the shades of red. Detailed information on the color of the sap in the CIE L * a * b * color space is shown in Figure 2.

### 3.2 Sensory analysis of the color of orange juices by the serialization method

The female students taking part in the survey were between 18 and 30 years old. The average age of the respondents was 22.69 ± 2.16.

When assessing the color of orange juice by glass, statistically significant differences were found in the respondents’ assessments (*p* < 0.05). In addition, the raters show poor agreement in their ratings because the Kendall coefficient of agreement is 0.13. The study showed that the group of 71 dieticians did not find significant differences in desirability between the samples of RJ, SN, SO, and ON juices. The VF and LI juice samples were different, with a significantly lower desirability than all other juice samples, but not statistically different and the least was LI juice (2.63 ± 1.55). Comparing these results to spectrophotometric measurements, RJ juice showed low brightness and a considerable approximation to the red and yellow colors compared to other juices. On the other hand, the LI juice was the most abnormal in comparison to the others and turned green and blue in color.

When assessing the color of orange juice using bottles without the original manufacturer’s label, statistically significant differences were also found in the respondents’ assessments (*p* < 0.05). Again, the raters showed poor agreement in their ratings (Kendall’s coefficient of agreement = 0.03). The study showed no statistically significant differences in color desirability between RJ and VF juices in a bottle without the original manufacturer’s label. The SN and SO juices were different, but they were not statistically different. LI and ON juices were statistically similar to the two groups mentioned. In the case of color evaluation using unlabeled bottles, SN juice (3.87 ± 1.87) was the most desirable and VF juice (3.27 ± 1.70) the least. Compared to the results of the color assessment by glass, the respondents chose a very light juice with a color closest to red as the best in comparison to other samples. The last place was a darker juice, closer to green and blue on the axis.

When assessing the color of orange juice using bottles with the original manufacturer’s label, no statistically significant differences were found in the respondents’ assessments (*p* > 0.05). Thus, no statistically significant differences between individual juices were found. In assessing the color through the bottle with the manufacturer’s label, the respondents again chose SN juice (3.69 ± 1.69) as the one with the most desirable color for orange juice. On the other hand, ON juice was rated the worst (3.37 ± 1.82). In terms of color parameters in the L * a * b * system, it does not stand out in particular from other juices. As in the case of the evaluation via unlabeled bottles, VF juice was ranked low again (3.38 ± 1.67).

A collective summary of the results of the assessment of the color of juices by the glass, a bottle without a label, and a bottle with a label are presented in Table 2.

**TABLE 2:** Summary of the results of the assessment of the color of juices by the glass, a bottle without a label, and a bottle with a label.

	Glass			Unlabeled bottle			Bottle with label		
**Name**	**Sum of ranks**	**Rank average (*$\bar{x}$* ± SD)**	Significance of differences (*a* = 0.05*)	**Sum of ranks**	**Rank average (*$\bar{x}$* ± SD)**	Significance of differences (*a* = 0.05*)	**Sum of ranks**	**Rank average (*$\bar{x}$* ± SD)**	Significance of differences (*a* = 0.05*)
RJ	292	4.11 ± 1.47	b	232	3.27 ± 1.70	a	259	3.65 ± 1.79	a
VF	190	2.68 ± 1.76	a	218	3.07 ± 1.50	a	240	3.38 ± 1.67	a
LI	187	2.63 ± 1.55	a	253	3.56 ± 1.25	ab	244	3.44 ± 1.69	a
SN	286	4.03 ± 2.12	b	275	3.87 ± 1.87	b	262	3.69 ± 1.69	a
SO	268	3.77 ± 1.56	b	274	3.86 ± 1.81	b	247	3.48 ± 1.63	a
ON	268	3.77 ± 0.93	b	239	3.37 ± 1.93	ab	239	3.37 ± 1.82	a

$\bar{x}$, average; SD, standard deviation. *Sums of ranks or rank averages marked with the same letters do not differ significantly.

**FIGURE 3:**
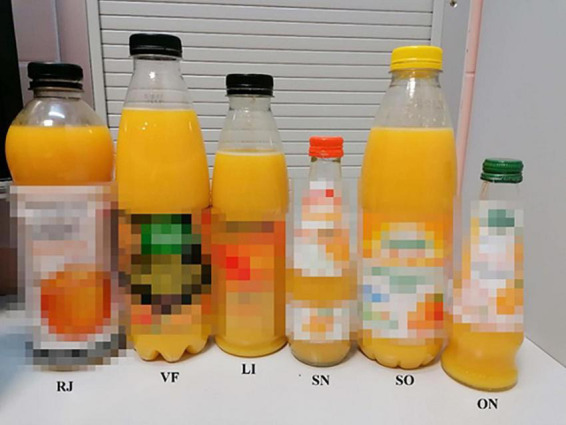
The design of the bottles.

Comparing the above results, the most desirable color of orange juice, when the original manufacturer’s packaging had no impact on the assessment, was RJ juice, and the least – was LI juice. RJ has anthropomorphic packaging with a white label and black cap. LI juice is round in shape with a transparent label and a black cap. During the evaluation by the original manufacturer’s bottle with and without a label, SN juice was selected for the best color juice, and VF juice was selected for the worst color. SN juice has a small, slim bottle with a white label and an orange cap. VF juice is round in shape with a predominantly black color (label and cap). Bottle designs are presented in Figure 3.

### 3.3 Correlation analysis between orange juice color evaluated using the CIELAB method and dieticians color evaluation by serialization method

A correlation analysis was conducted between dieticians’ evaluations of the color of the juice in the glass and color measurements in the CIELAB space. Pearson’s r correlation coefficients were used to determine whether there was a relationship between the variables. Detailed information is presented in Table 3.

**TABLE 3:** Ranks given to L*, a*, b* coordinate values and consumer preference assessment.

Juice name	L* (brightness)	a* (green-red tones)	b* (blue-yellow tones)	Consumer assessment
RJ	2	5	4	6
VF	1	2	2	2
LI	3	1	1	1
SN	5	6	5	5
SO	6	3	6	3
ON	4	4	3	4

The value of the correlation coefficient between dieticians’ preference and the L * coordinate was 0.14, suggesting that orange juice brightness (measured in the CIELAB space) has a moderately low positive correlation with dieticians’ visual evaluation. However, this correlation is not strong, meaning that changes in brightness are not strongly related to dieticians’ preference. The Pearson correlation coefficient value of 0.94 between dieticians’ preference and the a * coordinate indicates a strong positive correlation. This means that changes in the red-green component of the CIELAB color space are highly related to dieticians’ preferences. In practice, this may suggest that more intense colors in the red-green range may be preferred by dieticians. The Pearson correlation coefficient value of 0.6 between dieticians’ preference and the b * coordinate indicates a moderate positive correlation. This means that changes in the yellow-blue component of the CIELAB color space are moderately related to dieticians’ preference. In practice, this could mean that certain shades of yellow and blue are more attractive to dieticians. In summary, Pearson’s correlation analysis between dieticians’ preference and individual coordinates of the CIELAB color space provides an understanding of which color components of orange juice are more relevant to consumer taste. A stronger correlation may suggest that a particular component has a greater influence on dieticians’ visual evaluation preferences.

## 4 Discussion

Due to the topic and characteristics of this research article, there are few similar studies and new scientific reports from the last 10 years. Available studies usually address the dietary choices and consumer preferences of specific groups of people, but the dietary preferences of dieticians are not a frequent subject of research.

In the Suwała (4) study, the colors of carrot juices were determined spectrophotometrically and the results were compared to consumer preferences. Color measurements were made in the CIELAB system and the assessment of consumer preferences was made using the 5-point method. As a result, it was concluded that the most desirable juice is the one with parameters L * = 24.98; a * = 38.76; b * = 42.89, which the human eye perceives as an intense orange color, with a hue tending more toward red than yellow. In our study, the juice with the most desirable color was the juice with the values L * = 43.32; a * = 0.47; b * = 15.91, where, similarly to the Suwała study, the values of the b * parameter are higher than the values of the a * parameters, but at the same time the value of the L * parameter is the highest among all tested juices. This may be helpful for producers of juices whose color is commonly considered to be orange (orange, carrot), as they may try to control the color during the technological process to adjust to the preferences of consumers. A similar study was carried out by Fernandez-Vazquez et al. (15), this time on orange juices from five different varieties of this fruit. They were freshly squeezed through a juicer, then frozen at −21°C until testing, and thawed at room temperature for 24 h. Color measurement values ranged between 56.48 and 60.66 for L *, 12.40 and 24.60 for a *, and 58.12 and 64.66 for b *. Similar results were also obtained by Melendez-Martinez et al. (16), who measured the color of juices from the Valencia orange variety. They were frozen and stored at −18 to −21°C until distributed. The results they obtained were L * = 63.23, a * = 16.18, and b * = 64.64, indicating a deep, intense orange color. Compared to our study, where the results were between 39.34 and 43.98 for L *, −1.47 and 0.47 for a *, and 12.79 and 17.77 for b *, it can be hypothesized that the method of juice production and its storage may influence the color differences. Moreover, the results probably indicate that pasteurized juices show a lower color intensity than freshly squeezed juices fixed by deep freezing.

In the study by Fernandez-Vazquez et al. (15), the consumer assessment of juices was also carried out and these results were compared to the values of instrumental measurements. It was concluded that the most desirable juice was the one with the most orange color (L * = 57.41; a * = 18.29; b * = 60.98) and the least yellowish juice among all the tested samples (L * = 60.66; a * = 12.40; b * = 64.66). In our study, similar results were obtained. If the manufacturer’s packaging was excluded, the respondents rated the dark orange juice best (L * = 39.87; a * = −0.41; b * = 15.83) and the worst assessment was the lighter juice falling into tones blue and green (L * = 40.92; a * = −1.47; b * = 12.79 ± 0.10). In the next two studies, the color of juice from the Valencia orange variety was measured. Sánchez-Moreno et al. (17) squeezed the juice through a domestic juicer and filtered it through steel sieves. The color measurement results obtained are L * = 34.76; a * = − 2.48; and b * = 35.37. On the other hand, Pérez-López et al. (18) tested freshly squeezed juice which was then subjected to a temperature of 98°C for 20 s and obtained the results of L * = 52.99; a * = 5.50; and b * = 33.83. Compared to our research, this is a significant difference in values despite the similar method of juice preservation (high-temperature pasteurization) and a similar research methodology. In the works mentioned earlier in this section, a higher value of parameters was found in freshly squeezed juices than in those given in thermal treatment. In this case, it is the other way around. This leaves the topic open for discussion and further research to clarify what might have contributed to this discrepancy in the results.

Color measurements using the spectrophotometric method are not only used to monitor food quality or adjust product characteristics to the preferences of consumers. Pérez-López et al. (18) investigated the effect of the addition of mandarin orange juice on the improvement of the quality and desirability of orange juice. Thanks to CIELAB measurements combined with consumer sensory analysis, the first result they obtained was that among 100% of respondents, the color of mandarin juice was more desirable than the color of orange juice. Following this lead, they investigated whether consumers saw a difference between pure orange juice and juice with an addition of 3%, 6%, 9%, and 10% mandarin juice. They found that the differences were noticeable. This shows that instrumental color measurement may be necessary to control food additives to obtain the most desirable color.

The obtained results indicate that the product packaging may influence the consumer’s assessment of the color. The color assessment using a glass was the most reliable because each juice was served in the same vessel. Nothing disturbed the judgment. In this case, the juice with the best color turned out to be RJ juice. However, when judging the color through the unlabeled original manufacturer’s bottle, this juice dropped significantly in the ranking. In both cases, i.e., the bottles without and with the original manufacturer’s label, the SN juice was rated the best. The difference can be found in the shape and color of the bottle. Color plays an important role in increasing sales. Red has been associated with excitement, but also with danger. In contrast, blue is often used to represent openness and a peaceful imagination. When considering emotional responses, light colors often evoke positive associations, while dark colors primarily evoke negative ones. Black, for example, causes negative emotions such as sadness, depression, fear, and anger, mainly because it is also associated with the concept of death. At the other end of the spectrum, bright colors such as red, orange, and yellow can cause euphoria. Among different perceptions, red has been shown to produce feelings of warmth and intimacy, while some other shades are irritating (19). As with color, it has been observed that the shape of the packaging, especially for bottles, also has a significant impact on the perception of the brand among consumers as it can communicate the apparent advantages and disadvantages of the product. In addition, consumers tend to judge product volume and comfort based on sight or touch, especially for food packaging, which helps to identify color, shape, and size as key dimensions of packaging design (20). Consumers perceive products in attractive packaging as having higher quality than in less attractive packaging. The design and color of the product can also be used as an indicator of the price range. More expensive products tend to have darker colors, while less expensive products tend to have lighter shades. Additionally, it has been shown that the shape of the package influences the perception of volume, for example, elongated shapes are seen as offering better value for money (21). The studies by Mehta et al. (22) showed that the expectations for the product (orange juice) formed during the first contact with the packaging affect the sensory experience, as consumers expected that the juice from the glass bottle would be fresher than from other packaging materials. According to Chittur et al. (21), the most important thing is the synergy between the shape and color of the bottle. The white color of the cap seemed to have the most positive effect compared to black, red, and blue. However, this hypothesis was only applicable to the combination of a black-capped round bottle, as the combination of a square bottle with a black cap had already obtained a much higher preference index. In our study, the most desirable juice was the one in a glass bottle with an elongated shape, with a white label with orange elements, and an orange cap. According to Wolniak and Zadura (23), respondents chose packages with warm and light (bright) colors. This is related to attracting attention. Moreover, warm colors stimulate the imagination and evoke a feeling of happiness. In this case, a small glass bottle could also have influenced consumers’ choices. It seems that this type of packaging is perceived as more luxurious, expensive, fresh, home-made, produced on a small scale, and more valuable. However, these are only the researcher’s own thoughts and are undoubtedly worth exploring in subsequent research (21).

However, the study has its limitations. The study focused only on orange juices, which may limit the generalizability of its results. Dieticians’ color preferences can vary widely depending on the type of juice. While the study analyzes the importance of color in product choice, it does not delve into other factors that may influence consumer choices, such as taste, price, or brand reputation. A more comprehensive analysis would provide a more nuanced understanding of consumer behavior. The sensory analysis included participants evaluating juices without the original label and with the label. This setting may not fully reflect the actual experiences of dieters, where branding and labeling play a significant role in product perception. In addition, only female dietitians between the ages of 18 and 30 participated in the study. Age and gender may be important determinants of sensory evaluation. The strengths of this study should also be mentioned. The study used a combination of objective and subjective methods to evaluate the color of selected juices, providing a comprehensive picture. The use of a spectrophotometer in the first stage ensured precise and unbiased color measurement. The inclusion of sensory analysis using a ranking method included consumer preferences, adding a practical aspect to the research. This approach can help bridge the gap between scientific measurements and actual consumer choices. The study found a clear preference among dieticians for bright juices with a vibrant orange hue. This finding could be valuable for product development and marketing strategies, as it underscores the importance of color in consumer choices. The study confirmed the influence of product packaging on consumer choices, highlighting the synergy between color and packaging elements. This recognition can guide product design and marketing efforts. The use of objective color measurement techniques, such as the L * a * b * parameters, increases the reproducibility of the study’s results, making it easier for other researchers to validate or use the results. It should be mentioned that there are no other comparative studies that assess the consumer preferences of dietitians and this is a group that influences and shapes the public’s dietary choices.

## 5 Conclusion

Based on spectrophotometric measurements, there was significant variation in the color parameters of orange juices. The results of sensory tests indicate that dieticians are more likely to reach for bright juices with an intense orange hue. In addition, the product’s packaging influences dieticians’ dietary choices regardless of its content. When choosing orange juice, the synergy between packaging elements is also important. Instrumental color control during product manufacturing is justified, as well as the selection of packaging elements to achieve an attractive synergy.

## Data availability statement

The raw data supporting the conclusions of this article will be made available by the authors, without undue reservation.

## Ethics statement

The studies involving humans were approved by the Komisja Bioetyczna przy Śla̧skim Uniwersytecie Medycznym w Katowicach. The studies were conducted in accordance with the local legislation and institutional requirements. The participants provided their written informed consent to participate in this study. Written informed consent was obtained from the individual(s) for the publication of any potentially identifiable images or data included in this article.

## Author contributions

MK: Methodology, Project administration, Writing – review & editing, Funding acquisition. AK: Resources, Writing – original draft. KS: Conceptualization, Data curation, Methodology, Writing – review & editing. BS: Formal Analysis, Software, Writing – review & editing. WS-B: Validation, Visualization, Writing – review & editing. AB: Investigation, Supervision, Writing – original draft.
